# α2-Antiplasmin as a Potential Therapeutic Target for Systemic Sclerosis

**DOI:** 10.3390/life12030396

**Published:** 2022-03-09

**Authors:** Yosuke Kanno, En Shu

**Affiliations:** 1 Department of Clinical Pathological Biochemistry, Faculty of Pharmaceutical Science, Doshisha Women’s College of Liberal Arts, 97-1 Kodo Kyotanabe, Kyoto 610-0395, Japan; 2 Department of Dermatology, Graduate School of Medicine, Gifu University, 1-1 Yanagido, Gifu 501-1194, Japan; shu1402@gifu-u.ac.jp

**Keywords:** systemic sclerosis, α2-antiplasmin, fibrosis, vascular damage, fibrinolysis

## Abstract

Systemic sclerosis is a connective tissue disease of unknown origin that is characterized by immune system abnormalities, vascular damage, and extensive fibrosis of the skin and visceral organs. α2-antiplasmin is known to be the main plasmin inhibitor and has various functions such as cell differentiation and cytokine production, as well as the regulation of the maintenance of the immune system, endothelial homeostasis, and extracellular matrix metabolism. The expression of α2-antiplasmin is elevated in dermal fibroblasts from systemic sclerosis patients, and the blockade of α2-antiplasmin suppresses fibrosis progression and vascular dysfunction in systemic sclerosis model mice. α2-antiplasmin may have promise as a potential therapeutic target for systemic sclerosis. This review considers the role of α2-antiplasmin in the progression of systemic sclerosis.

## 1. Introduction

Systemic sclerosis (SSc) is an autoimmune rheumatic disease of an unknown origin characterized by immune abnormalities, vascular damage, and fibrosis of the skin and visceral organs [1]. This process usually occurs over many months or years and can lead to organ damage or death. The precise mechanism of SSc progression remains unclear, and there are no therapies to halt the progression of the disease.

**Figure 1:**
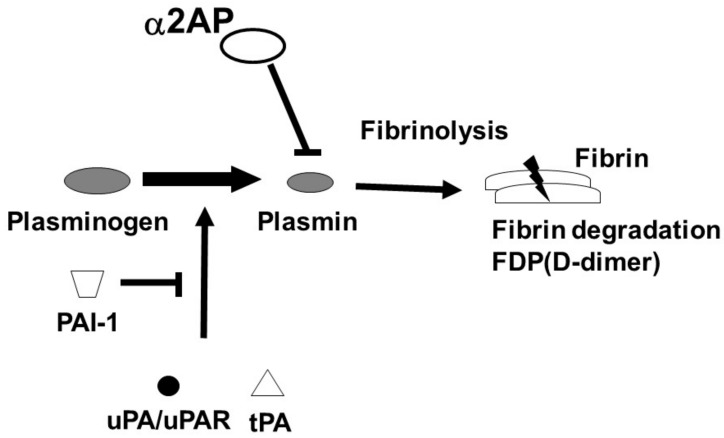
Fibrinolytic system. The fibrinolytic system dissolves fibrin. Plg is converted into plasmin by tPA or uPA/uPAR. The converted-plasmin digests fibrin clots, and FDP (D-dimer) is released into the bloodstream. α2AP functions as the main inhibitor of plasmin and inhibits fibrinolysis. PAI-1 binds and blocks tPA and uPA and inhibits the conversion of Plg to plasmin.

The fibrinolytic system dissolves fibrin and maintains vascular homeostasis. The regulators of fibrinolysis contain plasminogen (Plg), a proenzyme which is converted into plasmin by urokinase-type PA (uPA)/uPA receptor (uPAR) or tissue-type plasminogen activator (tPA). The converted-plasmin digests fibrin clots, and fibrin degradation products (FDP) of different molecular weights, including D-dimer, are released into the bloodstream. In contrast, α2-antiplasmin (α2AP) functions as the main inhibitor of plasmin, forming a stable complex plasmin-α2AP (PAP), and results in the inhibition of fibrinolysis [2] (Figure 1). Plasminogen activator inhibitor-1 (PAI-1) binds tPA and uPA and inhibits the generation of plasmin. It has been reported that an uPAR deficiency promotes endothelial dysfunction and fibrosis progression [3,4], and a α2AP deficiency and PAI-1 neutralization attenuate dermal inflammation and fibrosis progression in the bleomycin-induced SSc model mice, and multiple studies suggest that the fibrinolytic factors are associated with the pathology of SSc [5,6,7].

α2AP is a serine protease inhibitor (serpin) that rapidly inactivates plasmin on the fibrin clots or in the circulation [2,8,9]. α2AP has various biological functions independent of plasmin and is associated with thrombosis, angiogenesis, vascular remodeling, fibrosis, brain functions, and bone homeostasis [10,11,12,13,14,15,16,17]. The expression of α2AP is elevated in SSc dermal fibroblasts, and the blockade of α2AP suppresses the progression of pathology in SSc dermal fibroblasts and SSc model mice [18,19]. This review describes the biological functions of α2AP and summarizes the role of α2AP in the progression of SSc.

## 2. Systemic Sclerosis

SSc is a connective tissue disease of unknown origin characterized by the fibrosis of skin and visceral organs and peripheral circulatory disturbance. The progression of SSc is associated with immune abnormalities (immune cell activation and auto-antibodies production), vascular dysfunction (defective angiogenesis and vasculogenesis, vascular tone alteration, coagulation abnormalities, and endothelial to mesenchymal transition (EndoMT)), and fibrosis (extracellular matrix (ECM) over-production and ECM degradation inhibition) [1]. These abnormalities occur in the various stages of the disease, and these features influence each other and lead to extensive fibrosis and involvement of multiple organs [20,21]. However, the progression of this disease is not completely understood, and there are no therapies to halt the progression of this disease.

## 3. α2AP

**Figure 2:**
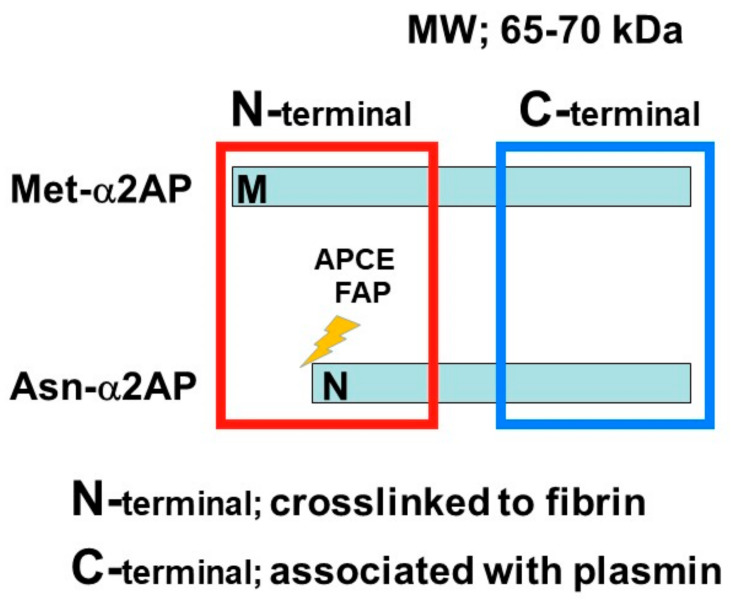
α2AP α2AP is a serpin with a molecular weight of 65–70 kd. The N-terminal sequence is crosslinked to fibrin, and the C-terminal region regulates the interaction with plasmin. Antiplasmin-cleaving enzyme (APCE) or fibroblast activation protein (FAP) causes the cleaving of Met-α2AP to Asn-α2AP (12-amino-acid residue shorter form).

α2AP is a serpin with a molecular weight of 65–70 kd [2] and functions as the main inhibitor of plasmin, a principal component of the fibrinolytic system [2,8,9]. α2AP has been observed in a number of tissues, such as the liver and kidney [22]. Congenital deficiency of α2AP is inherited in an autosomal recessive condition, and individuals with a homozygous α2AP deficiency exhibit severe bleeding symptoms, while heterozygous individuals have mild bleeding tendencies or may be asymptomatic [23,24]. It has been reported that α2AP is associated with pulmonary embolism, ischemic stroke, thrombotic thrombocytopenic purpura (TTP), and arterial thrombosis, and the removal of venous thrombi, wound healing, and fibrosis in several animal studies [6,23,25]. The N-terminal sequence is crosslinked to fibrin by Factor XIIIa (FXIIIa), and the C-terminal region regulates the interaction with plasmin. An antiplasmin-cleaving enzyme (APCE) or fibroblast activation protein (FAP), such as dipeptidyl peptidase 4 (DPP4), causes the cleaving of Met-α2AP to Asn-α2AP (12-amino-acid residue shorter form) [26,27]. It has been reported that Asn-α2AP becomes cross-linked to fibrin approximately 13 times faster than Met-α2AP during clot formation (Figure 2) [28]. In addition, matrix metalloproteinases-3 (MMP-3) inactivates α2AP by cleaving its Pro19-Leu20 peptide bond [29].

**Figure 3:**
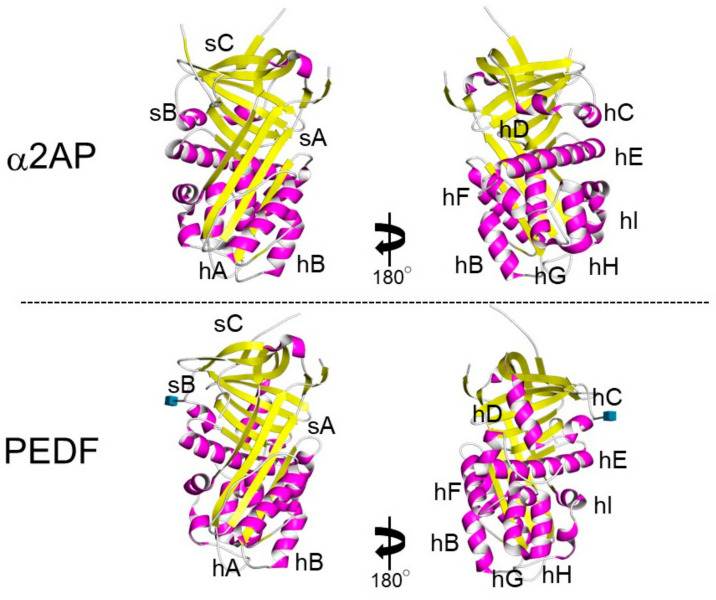
The structure of α2AP and PEDF. The structures of α2AP and PEDF are based on 2R9Y.pdb and 1IMV.pdb, respectively. The three β-sheets are shown in yellow (labeled sA-sC) and the 9 α-helices are shown in pink (labeled hA-hI).

α2AP is most closely related to the noninhibitory serpin pigment epithelium-derived factor (PEDF) [30]. Both α2AP and PEDF have three β-sheets and nine α-helices, and their positions are similar (Figure 3) [31,32]. α2AP and PEDF have very similar structures, and α2AP can bind and activate adipose triglyceride lipase (ATGL), which is known to be a receptor for PEDF [33] and regulates cell signaling, cytokine production, ECM production, cell differentiation, and cell proliferation [5,10,11,34,35]. PEDF has been shown to cause anti-angiogenic effects by inhibiting VEGF signaling [36] and α2AP also inhibits VEGF signaling [19]. α2AP and PEDF may bind the same protein and have similar functions. Furthermore, α2AP contains an arginine-glycine-aspartic acid (RGD) sequence, which is a recognition sequence for integrins [37].

## 4. α2AP Intracellular Signaling

α2AP can activate multiple intracellular signaling pathways, such as c-Jun N-terminal kinase (JNK), extracellular signal-regulated kinase 1/2 (ERK1/2), p38 mitogen-activated protein kinase (MAPK), and src-homology domain-2, containing tyrosine phosphatase 2 (SHP2) [11,13,19,38,39]. In addition, α2AP affects the vascular endothelial growth factor (VEGF) signaling [19], the angiotensin II (AngII) signaling [15], and the advanced glycation end products (AGEs)-induced smad signaling [40]. α2AP and PEDF have a similar structure, and α2AP can activate the adipose triglyceride lipase (ATGL), which is one of the PEDF receptors. PEDF can bind to ATGL, laminin receptor (LR), low-density lipoprotein-related protein 6 (LRP6), and the Notch receptor, and activate various cell signal pathways such as JNK and p38 MAPK [36]. α2AP activates phospholipase A_2_ (PLA_2_) through ATGL, which then promotes prostaglandin F_2α_ (PGF_2α_) synthesis and transforming growth factor-β (TGF-β) production [34]. In addition, α2AP deficiency promotes the expression status of β-catenin, and α2AP attenuates Wnt-3a-induced β-catenin expression and LRP6 activation [12]. Thus, α2AP can activate cell signaling through PEDF-binding proteins, such as ATGL and LRP6, and α2AP may also bind other PEDF-binding proteins and activate various cell signal pathways. On the other hand, α2AP has an RGD sequence at its C-terminus [37], and the RGD sequence affects cell recognition and platelet activation through integrin signaling [41,42].

**Figure 4:**
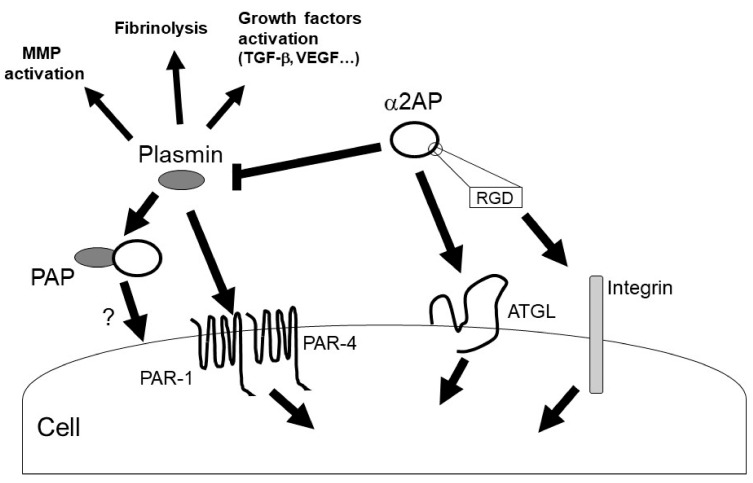
α2AP signaling. α2AP rapidly inactivates plasmin in fibrin clots or in the circulation, resulting in the formation of PAP. α2AP not only inhibits plasmin activity but also activates ATGL and regulates cell signaling. In addition, α2AP contains an RGD sequence and regulates integrin signaling. On the other hand, plasmin has various functions such as fibrinolysis, growth factors and MMPs activation.

Plasmin regulates the various signal pathways such as ERK1/2, p38 MAPK, Akt nuclear factor-κB (NF-κB), adenosine monophosphate-activated protein kinase (AMPK), signal transducers, and activators of transcription (STAT) pathways [9,43,44,45]. In addition, plasmin can activate growth factors such as TGF-β, VEGF, basic fibroblast growth factor (bFGF), pro-brain derived neurotrophic factor (proBDNF), insulin-like growth factor-binding protein 5 (IGFBP-5), and hepatocyte growth factor (HGF) [31,46,47]. Furthermore, plasmin can activate MMP-1, MMP-3, MMP-9, and protease-activated receptor-1 (PAR-1), PAR-4, platelets, factors V, VIII, and X [9,48,49,50]. α2AP may regulate various biological functions through plasmin inhibition and α2AP’s self-mediated signaling (Figure 4).

## 5. α2AP Deposition in SSc

The expression of α2AP is elevated in dermal fibroblasts obtained from SSc patients and in the fibrotic tissue of SSc model mice [11,18]. In addition, the levels of PAP in plasma are elevated in patients with SSc [51]. The deposition of α2AP may affect the progression of SSc.

Connective tissue growth factor (CTGF) and interferon-γ (IFN-γ) induce α2AP production through the ERK1/2 and JNK pathways in fibroblasts [11]. In addition, high-mobility group box 1 (HMGB1) induced the production of α2AP through the receptor for advanced glycation end products (RAGE) in fibroblasts [11]. Serum CTGF, IFN-γ, and HMGB1 levels in SSc patients are higher than those in healthy controls [52,53,54]. The blockade of these factors by neutralizing antibodies or inhibitors attenuates dermal fibrosis in bleomycin-induced SSc model mice [54,55]. The increase in these factors may cause the induction of α2AP expression and be associated with the pathogenesis of SSc.

The cleavage of α2AP by MMP-3 inactivates α2AP functions [29,56]. Serum levels of anti-MMP-3 autoantibody and MMP-3 inhibitor, tissue inhibitors of metalloproteinase-1 (TIMP-1) are elevated in SSc patients [57,58]. In addition, the ratio of MMP-3/TIMP-1 is decreased in SSc dermal fibroblasts [56]. The decrease in MMP-3 activity by MMP-3 autoantibody and inhibitors in SSc may suppress α2AP degradation and cause α2AP deposition.

## 6. α2AP and Immune Abnormalities in SSc

Immune cells such as T cells, B cells, and macrophages have been found in the skin and blood of SSc patients and SSc model mice [59,60,61], and these immune cells have often been observed preceding the fibrotic process [62]. In SSc, B cells cause the production of autoantibodies and the secretion of pro-inflammatory and pro-fibrotic cytokines such as TGF-β, tumor necrosis factor-α (TNF-α), and interleukin-6 (IL-6). B cells also cooperate with fibroblasts, endothelial cells (ECs), and T cells [61], and B cells are associated with EC apoptosis, fibroblast activation, the upregulation of type I collagen synthesis, and regulate the progression of fibrosis and vascular dysfunction in SSc [63,64]. T cells produce various cytokines such as IL-4, IL-5, IL-6, IL-10, and IL-13 [64], and T cell-produced cytokines regulate macrophage activation. In contrast, dermal fibrosis progression is induced in the bleomycin-administrated T and B cell-deficient severe combined immune deficiency (SCID) mice [65,66]. T cells contribute to macrophage activation, but T and B cells may not be essential for the development of fibrosis. Classically (M1) and alternatively (M2) activated macrophages induce pro-fibrotic cytokines such as TGF-β and IL-6 and TIMPs production, fibroblast activation, and collagen production and collagen deposition, and macrophages play a pivotal role in the development of fibrosis in SSc [63,67]. In particular, M2 macrophages are elevated in SSc patients [68]. M2 macrophages are known to be induced by IL-4 and IL-13 [69], and the blockade of IL-4 and IL-13 signaling by IL-4Rα neutralizing antibodies attenuates the progression of fibrosis in SSc model mice [66]. The increase in pro-fibrotic cytokine expression caused by these immune cells is associated with myofibroblast conversion from tissue-resident fibroblast and bone marrow-derived mesenchymal stem cells (MSC), epithelial-to-mesenchymal transition (EMT), and EndoMT. Myofibroblast deposition subsequently promotes excessive ECM production [70,71]. On the other hand, autoantibodies such as anti-MMP-1, anti-MMP-3, and anti-fibrin bound tPA antibodies have been identified in SSc patients, and TIMPs are elevated in SSc [58,72,73,74,75,76], and these factors suppress ECM degradation. Over-production and suppressed degradation of ECM cause fibrosis. Thus, these immune cells are associated with the overproduction of ECM and suppression of ECM degradation and have a major role in the onset of fibrosis in SSc. The correlation between fibrosis progression and the existence of specific autoantibodies such as anti-centromere antibodies and anti-Scl70 antibodies in SSc patients is unknown.

α2AP induces inflammatory cytokine production such as IL-1β and TNF-α [38,39], and α2AP deficiency affects neutrophil recruitment, lymphocyte infiltration, and IgE production [16,77,78]. In addition, PAP causes an increase in IgG and IgM secretion [79]. The blockade of α2AP by α2AP neutralizing antibodies attenuates anti-Scl70 antibody production in SSc model mice [18]. On the other hand, plasmin directly and indirectly regulates cell migration, cell proliferation, cell adhesion, monocyte chemotaxis, macrophage phagocytosis, neutrophil aggregation, monocyte/macrophage infiltration into tissues, and the release of cytokines, growth factors, and other inflammatory mediators [43,80,81,82,83,84,85,86]. In addition, plasmin can activate protease-activated receptor (PAR), platelets, factors V, VIII, and X, and mediate inflammation response [9,48,49]. Furthermore, plasmin effectively cleaves complement factors C3 and C5, thereby releasing the respective chemotactic anaphylatoxin fragments [87], which results in potentiation of TLR4 signaling [88]. α2AP contributes to inflammatory response, immune modulation, antibody production, and plasmin inhibition, and may play an important role as a mediator of inflammation and the immune system in SSc.

## 7. α2AP and Vascular Damage in SSc

Vascular damage in SSc includes morphological and functional changes. Microvascular disorders, including Raynaud’s phenomenon, telangiectasias, and digital ulcers, frequently occur in SSc patients [21,89,90]. Raynaud’s phenomenon is usually the initial manifestation and is found in more than 90% of SSc patients. Capillary microscopy of the nailfold of the fingers is a good tool to observe abnormalities in microvasculature. In the active stage of the disease, moderate capillary loss and enlargement, as well as microhemorrhages, are observed. In longstanding SSc, luminal constriction and obstruction are observed as a result of intimal proliferation and marked fibrosis. Furthermore, besides microvascular disorders, SSc patients have higher peripheral vascular disease, the prevalence of coronary atherosclerosis, and cerebrovascular calcification [91].

A number of factors, such as autoantibodies and inflammation caused by immune abnormalities, induce various cytokine production, persistent EC activation, impairment of cell-cell adhesion, EC apoptosis, and the activation of complement and coagulant pathways, and cause the progression of vascular damage, defective angiogenesis and vasculogenesis, EndoMT, vascular tone alteration, and coagulation abnormalities [20,92]. Furthermore, EC damage induces platelet activation, and the activated platelets release profibrotic factors such as TGF-β and platelet-derived growth factor (PDGF). These factors induce fibroblast activation, ECM production, and reactive oxygen species (ROS) release [93]. The activated platelets also activate T cells and B cells through serotonin and CD40L release [93,94]. There is an imbalance between vasodilation and vasoconstriction in SSc. The decrease in endothelial nitric oxide synthase (eNOS) expression and nitric oxide (NO) release from ECs attenuates vasodilation. In the meantime, the increase in vasoconstrictors such as endothelin-1 (ET-1) accelerates abnormal vasoconstriction [63]. Although the expression of VEGF is elevated in the patient’s skin of SSc, defective angiogenesis is evident. Moreover, a decrease in the number of endothelial progenitor cells (EPC) has been reported in SSc patients, resulting in compromised vasculogenesis. Defective angiogenesis and vasculogenesis then cause capillary loss and fibrosis. In SSc, vascular dysfunction is also observed in the lung, kidney, and other organs as well as the skin, causing the development of pulmonary arterial hypertension (PAH) and kidney manifestation [21]. Thus, vascular dysfunction causes more immune abnormalities and ECM deposition in SSc.

α2AP induces the reduction of blood vessels and blood flow and causes vascular damage in mice [19]. In addition, α2AP is associated with vascular remodeling, EC apoptosis, VEGF production, and angiogenesis [10,15]. α2AP deficiency causes VEGF overproduction and an increase in angiogenesis in the cutaneous wound healing process [10], and α2AP attenuates the VEGF-induced pro-angiogenic effects such as EC proliferation and tube formation by inhibiting VEGFR2 through SHP2 activation [19]. The expression of VEGF is elevated in various types of different cells, such as immune cells, ECs, and fibroblasts; nevertheless, vascular insufficiency manifests in SSc [95,96]. The increase in α2AP may cause the impairment of VEGF responses in SSc. In addition, AngII, which regulates vascular constriction and increases blood pressure [97], has profibrotic activity, and serum AngII levels in patients with SSc are higher than those in healthy control [98]. α2AP deficiency attenuates the AngII-induced vascular remodeling and perivascular fibrosis [15]. The increase in α2AP may promote AngII signaling in SSc. Furthermore, α2AP positively regulates the AGEs-induced EndoMT progression through smad signaling activation [40]. The accumulation of AGEs has been observed in the skin of SSc patients [99,100], and the combination of AGEs and α2AP may affect EndoMT progression in SSc. 

Plasmin damages EC integrity and endothelial barrier function and causes EC injury [101,102]. In addition, plasmin regulates fibrin-mediated EC spread and proliferation [103], platelet activation and platelet release reactions through PAR [49,104,105], MMP-regulated cell adhesion and cell migration [106], and TGF-β-mediated EC apoptosis [107]. Furthermore, plasmin regulates osteoprotegerin (OPG) production through the ERK1/2 and p38 MAPK pathways [43]. OPG is elevated in SSc and is associated with vascular calcification and atherosclerosis in SSc patients [108]. α2AP plays an important role in vascular homeostasis through its functions and plasmin inhibition and may affect the progression of vascular dysfunction in SSc.

## 8. α2AP and Fibrosis in SSc

Fibrosis is defined by tissue overgrowth, hardening, and/or scarring due to excessive production, deposition, and contraction of ECM. This process usually occurs over many months or years and causes organ damage or death. The progression of fibrosis is considered to result from maladaptive repair processes caused by profibrotic factors such as TGF-β, CTGF, AngII, PDGF, IFN-γ, and HMGB1, and the profibrotic factors induce myofibroblast differentiation from tissue-resident fibroblasts and bone marrow-derived mesenchymal stem cells (MSCs), epithelial-to-mesenchymal transition (EMT) and EndoMT [55,70,71,109,110,111]. In addition, PGF_2α_ is elevated in SSc patients and is associated with fibrosis progression independently of TGF-β [112,113]. Activated myofibroblasts promote excessive ECM and various cytokine production. Furthermore, SSc myofibroblasts are less prone to undergoing apoptosis [109]. In SSc, the phosphoinositide 3-kinase (PI3K)/Akt pathway and c-Abl are increased, and these factors facilitate myofibroblast survival by inhibiting the activity of BAX [109]. On the other hand, PAP and TIMPs expression are elevated in SSc, and anti-MMP-1 and anti-MMP-3 autoantibodies have been identified in SSc patients [51,58,72,73,74,75,76]. The increase in TIMPs expression and the suppression of MMPs and plasmin activity attenuate ECM degradation and lead to ECM deposition. In addition, high oxidative stress biomarkers such as malondialdehyde (MDA), asymmetric dimethylarginine (ADMA), and 8-Isoprostane, in the circulating blood have been found in SSc patients to mediate the inflammatory response, EndoMT, and fibrosis progression [114,115,116].

α2AP deficiency attenuates fibrosis progression in the bleomycin-induced SSc model mice, and α2AP induces PGF_2α_ and TGF-β production through ATGL and is associated with myofibroblast differentiation, EMT, EndoMT, and ECM production [5,11,34,35,40]. In addition, profibrotic factors including CTGF, HMGB1 and IFN-γ induce α2AP production in fibroblasts [11,38,66], and the increase in α2AP expression may affect fibrosis progression in SSc. Furthermore, it has been reported that α2AP deficiency attenuates oxidative stress [117] and promotes apoptosis [17], and α2AP may affect the induction of oxidative stress and resistance to apoptosis in SSc. On the other hand, plasmin can activate MMPs, such as MMP-1, MMP-3, and MMP-9, and degrade ECM [118,119,120]. In addition, plasmin has an anti-fibrotic function through the activation of HGF, which contributes to anti-fibrosis [47,121] and the induction of myofibroblast apoptosis [122].

## 9. α2AP and Coagulation/Fibrinolysis in SSc

The fibrinolytic system is known to play an important role in the maintenance of vascular integrity. The fibrinolytic activity is impaired in SSc, and the impaired fibrinolysis system causes fibrin deposition and hyper-coagulation [123,124]. In addition, platelet activation and aggregation in association with the elevated levels of fibrinogen, von Willebrand factor (vWF), lysophosphatidic acid, and sphingosine-1-phosphate (S1P) have been observed in SSc patients [93,125,126]. Fibrin is the most abundant ECM protein during the initial stage of tissue repair and provides a provisional matrix, promoting inward migration of tissue repair cells and preventing excessive blood loss [127]. Fibrin is also associated with angiogenesis, re-epithelialization, fibroblast migration and proliferation, and wound contraction. The fibrin deposition affects vascular injury, inflammation, immune cell activation, fibroblast growth and migration, and contributes to tissue remodeling, thrombosis, hyper-coagulation, pulmonary hypertension, and inflammatory response through multiple mechanisms [128,129,130,131,132]. Furthermore, fibrin regulates leukocyte migration and cell-to-cell adhesion between leukocytes and endothelium, immune cell activation, phagocytosis, and cytokine production [128,133,134,135]. Persistent fibrin enhances collagen accumulation and is associated with the development of dermal fibrosis [127]. Fibrin functions as a ligand for toll-like receptor-4 (TLR-4), vascular endothelial-cadherin (VE-cadherin), intercellular adhesion molecule (ICAM)-1, α_5_β_1_, α_x_β_2_, α_M_β_2_ integrin, and stimulates various cells, including leukocytes, ECs, platelets, and fibroblasts [128,136,137,138]. In addition, fibrin can anchor to the endothelial surface through the very-low-density lipoprotein receptor and promote leukocyte transmigration [139]. In vascular diseases, fibrin is present in normal arterial intima and atherosclerotic lesions [129]. In rheumatoid arthritis, fibrin becomes autoantigenic by the posttranslational modification, citrullination, and contributes to the inflammatory response through the TLR-4 pathway [129,140]. The crosslinking of fibrin by FXIII stabilizes fibrin clots, and the stable fibrin supports increased inflammatory cell adhesion, migration, and cytokine production [128,141]. FXIII is also known to induce angiogenesis and neovascularization through VEGFR-2 and αvβ3 integrin [142]. In addition, oxidative stress affects the cross-linking, branching, and height distribution of fibrin and significantly alters thrombus composition and architecture [143,144]. The impaired fibrinolysis system results in fibrin deposition in SSc [123,124]. Patients with SSc may be predisposed to thrombotic arterial complications and macrovascular impairment, which may also contribute to immune abnormalities, vascular damage, and fibrosis progression in SSc. Fibrin clearance may affect the onset of SSc. 

α2AP is an important physiological substrate of FAP, including DPP4 [145]. The expression of DPP4 is elevated in the fibrotic skin of SSc patients [146]. The increase in DPP4 expression in SSc may induce the conversion of Met-α2AP to Asn-α2AP and promote cross-linking to fibrin, which then causes impaired fibrinolysis and fibrin deposition in SSc.

## 10. The Role of α2AP as a Therapeutic Target for SSc

The treatment of α2AP neutralizing antibody improves vascular function and fibrosis and attenuates autoantibody production in SSc model mice [18,19]. In addition, the α2AP neutralizing antibody enhances fibrinolysis and thrombus dissolution [23,25,147]. Furthermore, α2AP deficiency attenuates oxidative stress [117], and α2AP neutralization may attenuate oxidative stress in SSc.

The inactivation of α2AP by MMP-3 recovers the pro-fibrotic phenotype of SSc dermal fibroblasts [56]. The treatment of MMP-3 may promote the degradation of α2AP and recovery of immune abnormalities, vascular damage, and fibrosis progression in SSc.

The inhibition of APCE causes the arrest of Met-α2AP conversion and results in increased fibrinolysis [148]. In addition, the inhibition of DPP4 exerts potent anti-fibrotic effects [146]. On the other hand, Plg interacts with DPP4 and regulates DPP4 activity [81]. The inhibition of APCE and DPP4 may attenuate the cleavage of α2AP and cause an increase in fibrinolysis. 

Small non-coding RNA sequences (miRNAs) are associated with vascular, immune response, and ECM homeostasis, and upregulation or downregulation of diverse miRNAs has been observed in blood and tissue from SSc patients [149]. MiR-29a represses α2AP and TIMP-1 expression and recovers the pro-fibrotic phenotype in SSc dermal fibroblasts [56,150]. The MMP-3 activation by the decrease in TIMP-1 expression may cause α2AP inactivation and affect the alleviation of SSc. In addition, an online database predicts that miR-30c can target α2AP mRNA, and the administration of miR-30c attenuates α2AP expression in the skin of SSc model mice [151]. MiR-30c also prevents pro-fibrotic changes such as myofibroblast differentiation, ECM overproduction, and vascular dysfunction, and exerts anti-fibrotic and anti-angiopathic effects in SSc model mice [151]. Furthermore, miR-133a-3p, miR-26a, and miR-30a attenuate fibrosis progression by inhibiting CTGF [152,153,154]. CTGF has been reported to induce α2AP expression [11]. This miRNA treatment may suppress α2AP expression.

The fusion protein of human serum albumin (HSA) to the α2AP N-terminal motif reduces fibrinolytic resistance by crosslinking to fibrinogen and fibrin [155]. In addition, synthetic peptide (AP26) corresponding to the carboxy-terminal region of α2AP enhances the conversion of plasminogen to plasmin induced by uPA and accelerates fibrinolysis [156]. The AP26 peptide also inhibits FXIIIa-catalyzed crosslinking of fibrin [156].

Microplasmin is a derivative of plasmin which lacks the five kringle domains [157], and microplasmin neutralize α2AP activity [158,159]. The neutralization of α2AP by microplasmin reduces ischemic stroke and improves neurological dysfunction [159,160]. 

**Figure 5:**
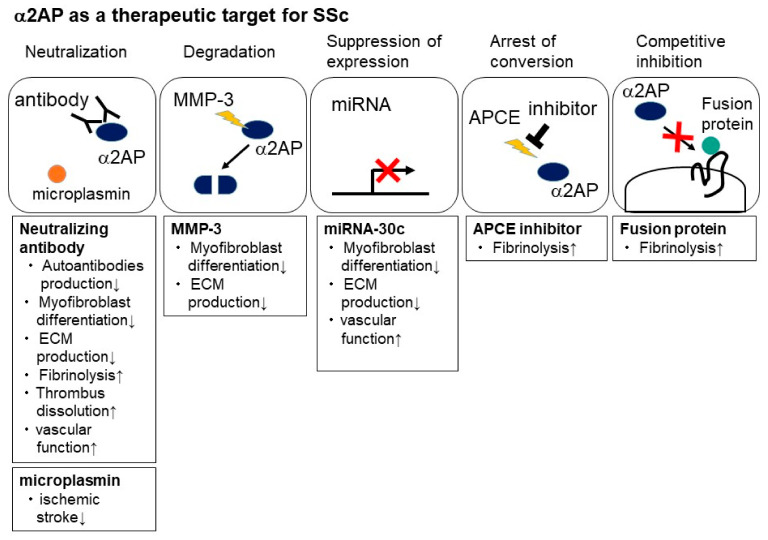
α2AP as a therapeutic target for SSc. The blockade and inactivation of α2AP by neutralizing antibody, MMP-3, APCE inhibitor, miRNA, fusion proteins, and microplasmin suppress myofibroblast differentiation and ECM production and enhance fibrinolysis and thrombus dissolution and may improve fibrosis progression and vascular dysfunction in SSc.

The blockade and inactivation of α2AP by neutralizing antibodies, MMP-3, APCE inhibitor, miRNA, fusion proteins, and microplasmin may improve fibrosis progression, vascular dysfunction, and impaired fibrinolysis in SSc (Figure 5).

## 11. Conclusions and Therapeutic Perspectives

**Figure 6:**
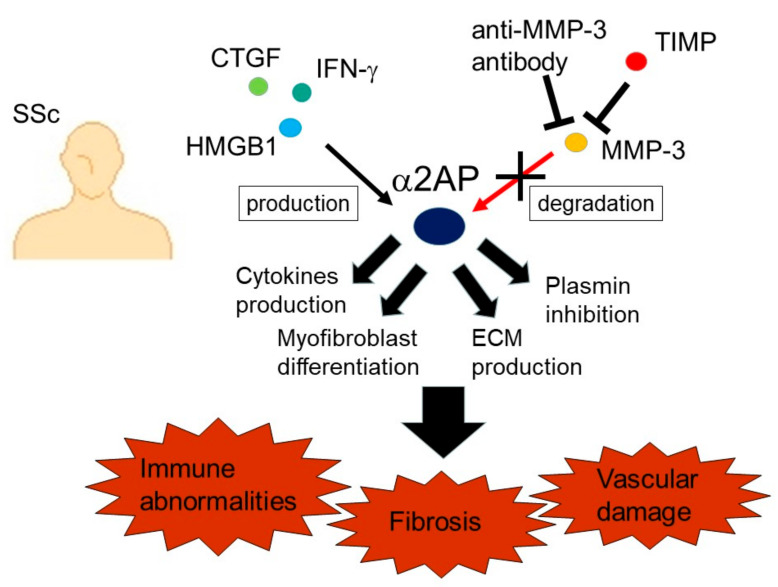
The mechanism of α2AP-associated SSc progression. The increase in these factors such as CTGF and HMGB1 induces α2AP expression, and the increase in anti-MMP-3 autoantibody and MMP-3 inhibitor, TIMP-1, inhibits α2AP degradation. α2AP deposition induces cytokine production, myofibroblast differentiation, ECM production, plasmin inhibition, and then may cause immune abnormalities, fibrosis progression, and vascular damage.

In SSc, immune abnormalities, vascular damage, and fibrosis contribute to disease progression. The changes in α2AP expression and activity may result in immune system activation, disruption of endothelial homeostasis, and aberrant ECM metabolism, which consequently contribute to SSc progression (Figure 6). The blockade of α2AP functions may prevent the progression of SSc and may be a novel therapeutic approach to SSc.

## Data Availability

Not applicable.
